# Frailty as a predictor of postoperative aspiration pneumonia after abdominal surgery for digestive cancers

**DOI:** 10.1097/MD.0000000000045697

**Published:** 2025-11-14

**Authors:** Shinichiro Yamada, Yuji Morine, Tetsuya Ikemoto, Yu Saito, Hiroki Teraoku, Mitsuo Shimada

**Affiliations:** a Department of Surgery, Tokushima University, Tokushima City, Tokushima, Japan.

**Keywords:** abdominal surgery, digestive cancers, frailty, malnutrition, postoperative aspiration pneumonia

## Abstract

This study was performed to investigate the factors associated with the development of postoperative aspiration pneumonia (PAP) after abdominal surgery for digestive cancers. The study involved 420 patients who had undergone abdominal surgery for digestive cancers. The patients were divided into a PAP group (n = 13) and non-PAP group (n = 407), and clinicopathological factors were compared between the 2 groups. Logistic regression analysis was used to assess the predictors of PAP, and develop a formula for the prediction of PAP. The PAP group showed a higher white blood cell count, C-reactive protein concentration, and modified Glasgow prognostic score and a lower albumin concentration than the non-PAP group (*P* < .05). The PAP group also showed a significantly higher rate of frailty and longer postoperative hospital stay (*P* < .05). The multivariate analysis revealed that cerebrovascular disease, respiratory complications, and frailty were independent predictive factors of PAP (*P* < .05). A prediction formula established using these factors showed a sensitivity of 92.3% and specificity of 76.4%. Evaluation of the preoperative frailty status is important for risk stratification and prevention of postoperative morbidity in patients undergoing surgery.

## 1. Introduction

The average life expectancy at birth has been increasing in many countries, with Japan having the highest life expectancy worldwide. This situation has led to a growing number of older patients being diagnosed with digestive cancers, presenting a significant global challenge in the management of malignancies among patients of advanced age.^[1]^ In particular, postoperative aspiration pneumonia (PAP) has become a critical issue. PAP is one of the most severe pulmonary complications, with mortality rates of up 38.5% following general and visceral surgeries.^[2,3]^ Furthermore, PAP increases the likelihood of reintubation, prolongs the hospital and intensive care unit stays, and increases costs.^[4,5]^ Identification of risk factors for PAP is therefore critically important. Factors such as older age, a history of pulmonary disease, and neurological disorders are known contributors.^[6,7]^ Procedure-related risk factors must also be considered, such as the type and duration of surgery and whether the procedure is an emergency operation.^[8]^ Despite these known risk factors, the optimal strategy for preventing PAP remains unclear.

In recent years, researchers have increasingly focused on the role of frailty—a multidimensional, heterogeneous syndrome associated with instability—in patients undergoing surgery.^[9]^ Frailty has an impact on postoperative morbidity, mortality, and functional decline.^[10]^ Many methods can be used to assess frailty, including the Fried frailty phenotype,^[11]^ the Study of Osteoporotic Fractures index,^[12]^ the FRAIL (fatigue, resistance, ambulation, illness, loss of weight) scale,^[13]^ and the modified Fried index.^[14]^ We have focused on the clinical frailty scale (CFS), a highly feasible and convenient 9-point global frailty scale involving evaluation of indices such as mobility, physical activity, and function; 1: very fit, 2: fit, 3: managing well, 4: living with very mild frailty, 5: living with frailty, 6: living with moderate frailty, 7: living with severe frailty, 8: living with very severe frailty, 9: terminally ill.^[15,16]^ We previously reported that frailty assessed using the CFS could predict the prognosis of patients undergoing surgery for pancreatic cancer and hepatocellular carcinoma.^[17]^ Moreover, frailty is associated with dysphagia because of the loss of muscle mass, and dysphagia due to frailty was recently recognized as a geriatric syndrome.^[18]^ Dysphagia is considered the most common cause of PAP, and one report discussed the impact of frailty on postoperative dysphagia in patients undergoing elective cardiovascular surgery.^[19]^ However, no report to date has focused on frailty and PAP after abdominal surgery for digestive cancers.

Therefore, we investigated the impact of frailty on PAP after abdominal surgery for digestive cancers and analyzed various predictive factors of PAP.

## 2. Methods

**Figure 1.**
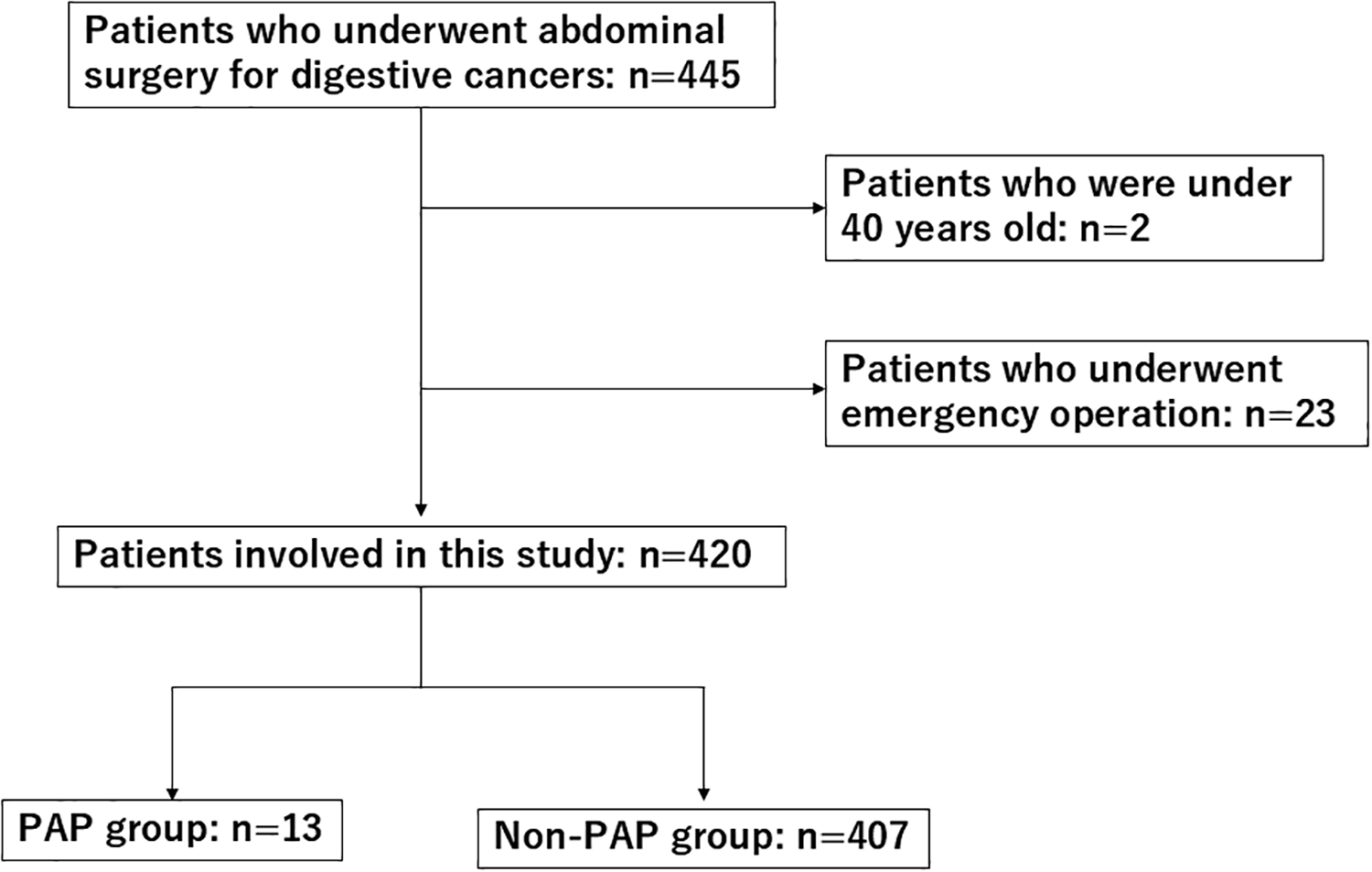
A flow chart for this study.

A total of 443 patients underwent abdominal surgery for digestive cancers at the Department of Surgery of Tokushima University Hospital from April 1994 to December 2022 were elected. Inclusion criteria were as follows: patients who underwent digestive elective surgery for digestive cancers; and patients over 40 years old. Exclusion criteria were as follows: patients who underwent emergency operation; and patients with preoperative or intraoperative aspiration pneumonia (e.g., micro-aspiration during intubation or prolonged ventilation, preoperative vomiting). Consequently, 420 patients were involved in this study (Fig. 1). The patients were divided into 2 groups: the PAP group (n = 13) and the non-PAP group (n = 407). Aspiration pneumonia was diagnosed by witnessed aspiration and subsequent confirmation by a conventional X-ray examination or computed tomography scan of the chest. The patients’ characteristics were collected from their medical records. The preoperative factors evaluated were age, sex, type of cancer, body mass index (BMI), and several blood test indices (white blood cell [WBC] count, total lymphocyte count, hemoglobin concentration, albumin concentration, C-reactive protein [CRP] concentration, neutrophil-to-lymphocyte ratio, and modified Glasgow prognostic score [mGPS]). The patients’ histories of respiratory complications, cerebrovascular disease, and frailty were also investigated. Frailty was defined as a CFS score of ≥4.^[17]^ The intraoperative and postoperative factors evaluated were the type of surgery (open or laparoscopic surgery), operation time, blood loss, and postoperative hospital stay. These factors were compared between the PAP and non-PAP groups. This study was approved by the ethics committee of Tokushima University Hospital (No. 3215), and the requirement for informed consent was waived. This study was conducted in accordance with Helsinki standards and STROBE criteria of retrospective studies.

### 2.1. Statistical analysis

We used the unpaired Mann–Whitney *U* test or the χ^2^ test to compare clinicopathological variables between the 2 groups. Numerical data were presented as median and range. Missing values are described in Tables 1 and 3 . Variables with a *P*-value of <.05 in the univariate analyses were included in the multivariate analysis using a logistic regression model. For all statistical analyses, *P* *<* .05 was considered significant. All statistical analyses were performed using JMP 8.0.1 statistical software (SAS Institute, Cary). Sample collection was opportunistic and sample size calculations were not performed.

## 3. Results

Of the 420 patients who underwent surgery for digestive cancers, 253 (61%) were male. The patients’ median age was 77 (41–95) years, and their median BMI was 22.5 (14.9–36.0) kg/m^2^. A total of 105 (25%) patients had frailty. The following surgical interventions were performed in the entire study population: gastrectomy (n = 112), hepatectomy (n = 101), pancreatic resection (n = 140), and colorectal resection (n = 67). Laparoscopic surgery was performed in 122 (29%) patients. According the inclusion criteria, all patients arrived in the normal ward postoperatively without a history of aspiration; a comparison was then performed between those who did and did not subsequently develop PAP.

**Figure 2.**
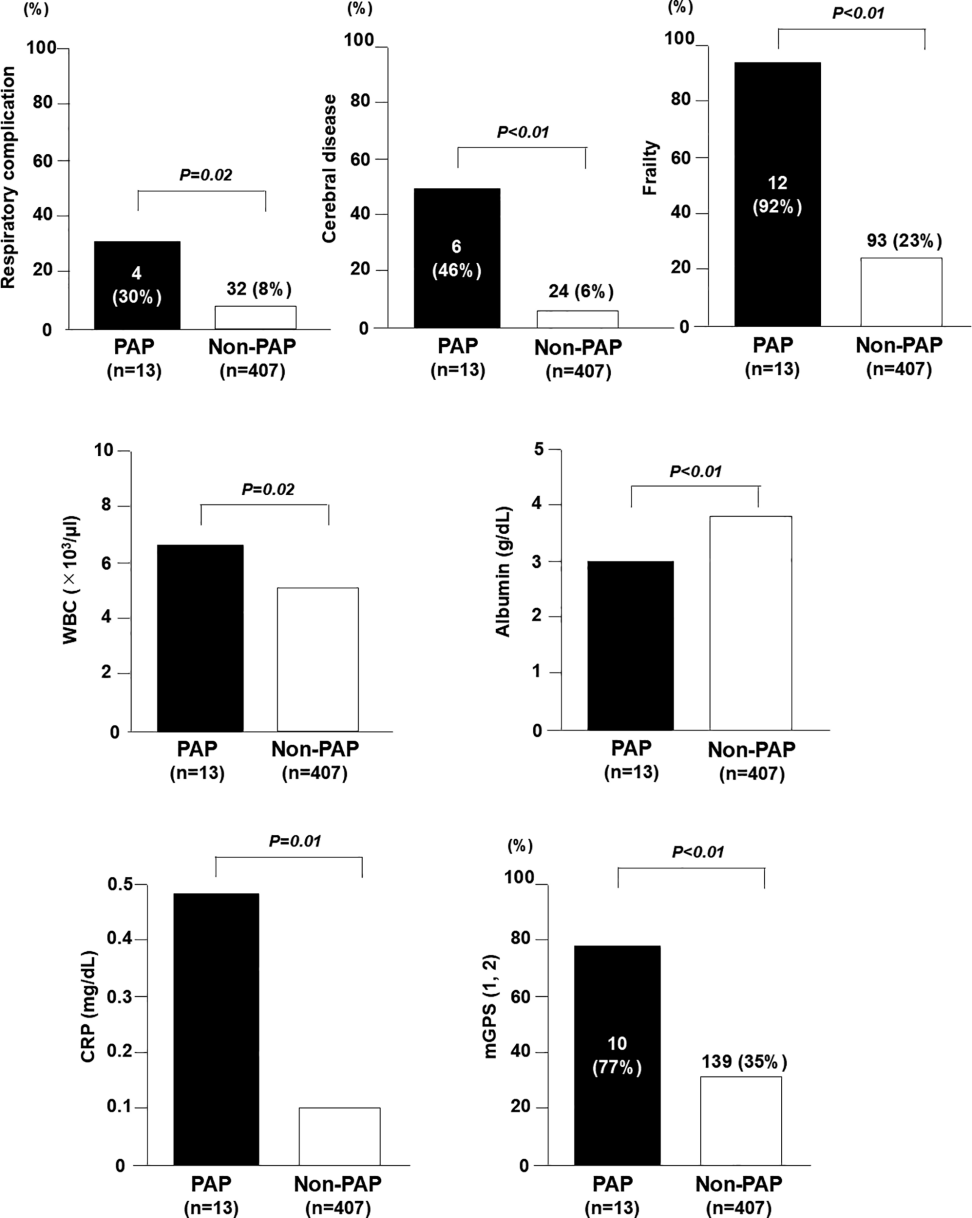
Bar graphs of factors which show significant differences between 2 groups. Patients in the PAP group showed significantly higher rates of cerebrovascular disease, respiratory disorder, and frailty (*P* < .01). Patients in the PAP group showed significantly higher level of WBC, CRP, mGPS and low albumin compared with non-PAP group (*P* < .05). CRP = C-reactive protein, mGPS = modified Glasgow prognostic score, PAP = postoperative aspiration pneumonia, WBC = white blood cell.

Table 1 summarizes the clinicopathological variables in the PAP and non-PAP groups. Patients in the PAP group showed a significantly higher WBC count, CRP concentration, and mGPS and a lower albumin concentration than patients in the non-PAP group (*P* < .05). Patients in the PAP group also showed significantly higher rates of cerebrovascular disease, respiratory complications, and frailty (*P* < .01). There was no significant difference in age, sex, BMI, type of cancer, surgical approach, operation time, or blood loss between the groups. The PAP group showed a significantly longer postoperative hospital stay (*P* < .01). Figure 2 presents a graph of the factors showing significant differences between the 2 groups.

**Table 1:** Patient characteristics in the PAP and non-PAP groups.

Variable	PAP (n = 13)	Non-PAP (n = 407)	*P*-value
Age (yr)	79 (59–91)	77 (41–95)	.26
Sex (male/ female)	11/ 2	247/ 160	.06
Procedure (stomach/ liver/ colon/ pancreas)	2/ 4/ 4/ 3	110/ 97/ 63/ 137	.40
Approach (open/ laparoscopy)	11/ 2	287/ 120	.24
BMI (kg/m^2^)	22.3 (18.7–25.9)	22.5 (14.9–36.0)	.89
Respiratory complication (+/ −)	4/ 9	32/ 375	.02
Cerebrovascular disease (+/ −)	6/ 7	24/ 383	<.01
WBC (/µL)	6300 (4200–13,000)	5400 (1800–13,600)	.02
TLC (/µL)	1430 (840–2350)	1140 (170–3800)	.51
Hemoglobin (g/dL)	11.9 (9.6–16.6)	12.8 (8.0–17.4)	.66
Albumin (g/dL)	3.0 (2.7–4.5)	3.9 (2.1–5.0)	<.01
CRP (mg/dL)	0.48 (0.05–3.58)	0.10 (0.05–11.3)	.01
NLR	2.99 (1.29–11.1)	2.73 (0.26–26.3)	.48
mGPS (0, 1/2)	8/ 5	358/ 43*	.01
Frailty (yes/no)	12/ 1	93/ 314	<.01
Operation time (min)	241 (121–589)	334 (138–758)	.06
Blood loss (mL)	124 (5–650)	160 (0–1600)	.56
Postoperative hospital stay (d)	59 (16–173)	20 (8–160)	<.01

Data are presented as median (range) or number of patients.

BMI = body mass index, CRP = C-reactive protein, mGPS = modified Glasgow prognostic score, NLR = neutrophil-to-lymphocyte ratio, PAP = postoperative aspiration pneumonia, TLC = total lymphocyte count, WBC = white blood cell.

* Missing values.

Table 2 shows the results of the multivariate analysis for aspiration pneumonia using logistic regression analysis. Among the 7 parameters confirmed in Table 1 (cerebrovascular disease, respiratory complications, WBC count, albumin concentration, CRP concentration, mGPS, and frailty), cerebrovascular disease, respiratory complications, and frailty were independent predictive factors of PAP (*P* < .05). Frailty was the best predictive factor of PAP (odds ratio = 40.9). Weighting these factors using logistic regression analysis, we devised the following prediction formula:

**Table 2:** Multivariate analysis of predictive factors of PAP.

Variable	OR (95% CI)	*P*-value
Respiratory complication (+ [Ref: –])	11.8 (1.55–90.1)	.02
Cerebrovascular disease (+ [Ref: –])	8.99 (1.91–42.3)	<.01
WBC (≥9000/µL [Ref: <9000])	5.53 (0.99–30.7)	.05
Albumin (≥4.0 g/dL [Ref: <4.0])	0.14 (0.01–2.09)	.15
CRP (≥0.3 mg/dL [Ref: <0.3])	0.51 (0.07–3.73)	.51
mGPS (0, 1 [Ref: 2])	5.17 (0.67–39.8)	.12
Frailty (yes [Ref: no)	40.9 (4.02–416.5)	<.01

CI = confidence interval, CRP = C-reactive protein, mGPS = modified Glasgow prognostic score, OR = odds ratio, PAP = postoperative aspiration pneumonia, WBC = white blood cell.


$$\begin{array}{lll} & 1.8\times \left(frailty\right)+1.1\times \left(respiratory\;complications\right)\; & +0.7\times \left(cerebrovascular\;disease\right) \end{array}$$

(for each factor, yes = 1 and no = 0)

**Figure 3.**
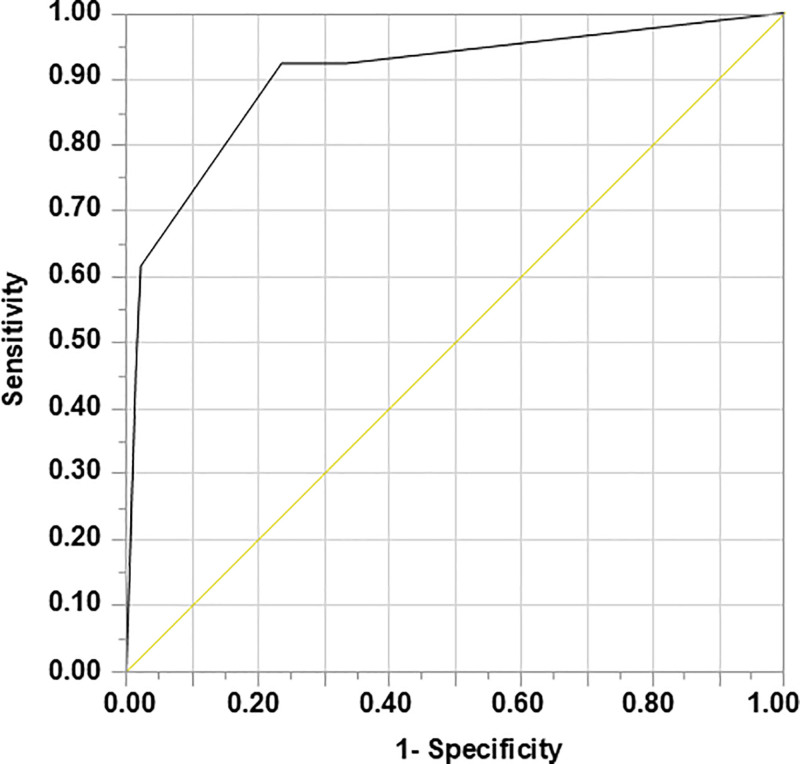
ROC curve of prediction formula for PAP. Cut off value 1.8 was useful for estimating PAP (sensitivity: 92.3%, specificity: 76.4%, AUC: 0.90). AUC = area under the curve, PAP = postoperative aspiration pneumonia.

A cutoff value of 1.8 was useful for estimating PAP (sensitivity = 92.3%, specificity = 76.4%, area under the curve = 0.90) (Fig. 3).

Among the 105 patients with frailty, 12 (11.4%) developed PAP. Various factors were compared between the PAP (n = 12) and non-PAP (n = 93) groups to identify those predictive of PAP in patients with frailty. Cerebrovascular disease, respiratory complications, WBC count, albumin concentration, CRP concentration, and mGPS were detected as predictive factors of PAP (Table 3). The multivariate analysis (Table 4) revealed that a high WBC count, cerebrovascular disease, and respiratory complications were independent predictive factors.

**Table 3:** Characteristics of patients with frailty in the PAP and non-PAP groups.

Variable	PAP (n = 12)	Non-PAP (n = 93)	*P*-value
Age (yr)	79.5 (59–91)	79 (57–95)	.72
Sex (male/female)	10/ 2	58/ 35	.13
Approach (open/laparoscopy)	10/ 2	76/ 17	.89
BMI (kg/m^2^)	22.0 (18.7–25.8)	22.0 (14.9–32.5)	.89
Respiratory complication (+/ −)	4/ 9	32/ 375	<.01
Cerebrovascular disease (+/ −)	4/ 8	5/ 88	<.01
WBC (/µL)	6350 (4700–13,000)	5800 (2100–13,600)	.046
TLC (/µL)	1415 (840–2356)	1226 (174–3547)	.22
Hemoglobin (g/dL)	11.9 (9.6–15.5)	12.8 (8.8–15.9)	.60
Albumin (g/dL)	3.1 (2.7–4.5)	3.7 (1.8–4.7)	.049
CRP (mg/dL)	0.52 (0.05–3.58)	0.16 (0.05–11.3)	.03
NLR	3.08 (1.85–11.1)	3.11 (0.70–26.3)	.94
mGPS (0, 1/ 2)	7/ 5	79/ 13*	.03
Blood loss (mL)	103 (5–532)	179 (0–980)	.31
Postoperative hospital stay (d)	47 (16–173)	20 (10–78)	<.01

Data are presented as median (range) or number of patients.

BMI = body mass index, CRP = C-reactive protein, mGPS = modified Glasgow prognostic score, NLR = neutrophil-to-lymphocyte ratio, PAP = postoperative aspiration pneumonia, TLC = total lymphocyte count, WBC = white blood cell.

* Missing values.

**Table 4:** Multivariate analysis of predictive factors of PAP in patients with frailty.

Variable	OR (95% CI)	*P*-value
WBC (≥9000/µL [Ref: <9000])	8.49 (1.20–59.8)	.03
Albumin (≥4.0g/dL [Ref: <4.0])	0.09 (0.003–2.48)	.16
CRP (≥0.3 mg/dL [Ref: <0.3])	0.56 (0.06–5.35)	.61
mGPS (0, 1 [Ref: 2])	6.21 (0.64–60.5)	.12
Respiratory complication (+ [Ref: –])	26.0 (1.92–352.4)	.01
Cerebrovascular disease (+ [Ref: –])	13.6 (2.27–82.0)	<.01

CI = confidence interval, CRP = C-reactive protein, mGPS = modified Glasgow prognostic score, OR = odds ratio, PAP = postoperative aspiration pneumonia, WBC = white blood cell.

## 4. Discussion

In this study, we demonstrated that cerebrovascular disease, respiratory complications, and frailty were independent factors that can be used to predict PAP, and frailty was the most effective predictive factor. Furthermore, a predictive formula was generated using these 3 independent factors. Although age,^[6]^ BMI, and surgical approach (open or laparoscopic)^[20]^ have been reported as risk factors, these factors were not identified as predictive factors in the present study.

Dysphagia is a common problem with potentially serious consequences such as malnutrition, dehydration, pneumonia, and death.^[21]^ Notably, dysphagia is the main cause of aspiration pneumonia. A meta-analysis revealed that frailty and prefrailty were associated with dysphagia and that frailty prevention was a necessary intervention for swallowing dysfunction.^[22]^ Moreover, the CFS, which was used to assess frailty in the present study, could be used to detect the risk of swallowing difficulty in older inpatients.^[23]^ One study showed that preoperative frailty was independently associated with postoperative dysphagia in patients undergoing elective cardiovascular surgery.^[19]^ To our knowledge, the current study is the first to show that the CFS can be used to predict PAP after abdominal surgery for digestive cancers, and it is the first to develop a predictive formula for PAP.

Factors detected in the univariate analysis, including a high WBC count, CRP concentration, and mGPS and a low albumin concentration, have been recognized as being associated with a high inflammatory and low malnutritional status. A high WBC count and CRP concentration accelerate the progression of frailty, and early identification of systemic inflammation is important to identify patients at high risk of frailty.^[24]^ A high CRP concentration has also been reported to be related to an acute inflammatory status and malnutrition.^[25]^ In fact, the patients with frailty in the present study showed a significantly higher WBC count, CRP concentration, mGPS, and neutrophil-to-lymphocyte ratio and a significantly lower albumin concentration than those without frailty (*P* < .05, data not shown).

Swallowing dysfunction has been shown to be particularly severe in malnourished patients,^[26]^ and inflammation can be used to estimate the outcome of patients with dysphagia.^[27]^ Based on these findings, frailty appears to be a good indicator of a high inflammatory and malnutritional status and therefore dysphagia.

Respiratory complications and cerebrovascular disease were also independent predictive factors of PAP in the present study. Furthermore, these factors are closely associated with frailty. Chronic obstructive pulmonary disorder has been shown to be a risk factor for incident pneumonia and death.^[28]^ Cerebrovascular disease is also a risk factor for aspiration pneumonia due to conditions such as post-stroke dysphagia.^[29]^ These factors are associated with frailty,^[30,31]^ and their prevalence further increased the risk of PAP in patients with frailty in our study.

Some studies have shown that rehabilitation and nutritional therapy are useful for improving dysphagia.^[32,33]^ Kagaya and Inamoto^[33]^ outlined various forms of resistance training as rehabilitation strategies for sarcopenic dysphagia, including the Shaker exercise, Mendelsohn maneuver, tongue-hold swallow exercise, jaw-opening exercise, swallow resistance exercise, lingual exercise, expiratory muscle strength training, neuromuscular electrical stimulation, and repetitive peripheral magnetic stimulation. Aggressive nutritional therapy combined with swallowing rehabilitation can further improve dysphagia. Energy intake of approximately 35 kcal/kg/day based on ideal body weight combined with swallowing rehabilitation has been recommended for sarcopenic dysphagia.^[32]^ Energy intake of ≥30 kcal/kg/day and protein intake of ≥1.2 g/kg/day based on ideal body weight increased tongue strength in older adults with sarcopenia.^[34]^ Furthermore, energy intake of ≥30 kcal/kg/day based on ideal body weight resulted in greater improvement of dysphagia than intake of <30 kcal/kg/day in patients with sarcopenic dysphagia.^[35]^

The present study has 2 main limitations. First, this was a retrospective study performed in a single center, and sample size of PAP patients in this study was small. Second, 4 types of cancers were included because the number of patients with each type of cancer was relatively small. Thus, further research with a larger prospective population is warranted to confirm our results.

In conclusion, frailty, respiratory complications, and cerebrovascular disease can be used to predict PAP after abdominal surgery for digestive cancers. Evaluation of the preoperative frailty status is important for risk stratification and prevention of PAP in patients undergoing surgery.

## Acknowledgments

We thank Angela Morben, DVM, ELS, from Edanz (https://jp.edanz.com/home) for editing a draft of this manuscript.

## Author contributions

**Conceptualization:** Shinichiro Yamada, Yuji Morine, Mitsuo Shimada.

**Data curation:** Shinichiro Yamada, Tetsuya Ikemoto, Yu Saito, Hiroki Teraoku.

**Investigation:** Shinichiro Yamada.

**Methodology:** Shinichiro Yamada, Tetsuya Ikemoto, Yu Saito, Hiroki Teraoku.

**Supervision:** Yuji Morine, Mitsuo Shimada.

**Writing – original draft:** Shinichiro Yamada.

**Writing – review & editing:** Yuji Morine, Mitsuo Shimada.
